# Promotion of osteoclastogenesis by IL-26 in rheumatoid arthritis

**DOI:** 10.1186/s13075-019-2070-0

**Published:** 2019-12-12

**Authors:** Kyung-Ann Lee, Kyoung-Woon Kim, Bo-Mi Kim, Ji-Yeon Won, Hong Ki Min, Dhong Won Lee, Hae-Rim Kim, Sang-Heon Lee

**Affiliations:** 1 Division of Rheumatology, Department of Internal Medicine, Research Institute of Medical Science, Konkuk University Medical Center, Konkuk University School of Medicine, Neungdong-ro 120-1, Gwangjin-gu, Seoul, 05030 South Korea; 2 Division of Rheumatology, Department of Internal Medicine, Soonchunhyang University Hospital, Seoul, South Korea; 3 Convergent Research Consortium for Immunologic Disease, Seoul St. Mary’s Hospital, College of Medicine, The Catholic University of Korea, Seoul, South Korea; 4 Department of Orthopaedic Surgery, Konkuk University Medical Center, Seoul, South Korea

**Keywords:** Rheumatoid arthritis, Interleukin-26, Osteoclastogenesis, RANKL

## Abstract

**Background:**

The inflammatory cascade in the rheumatoid arthritis (RA) synovium is modulated by a variety of cytokine and chemokine networks; however, the roles of IL-26, in RA pathogenesis, are poorly defined. Here, we investigated the functional role of interleukin-26 (IL)-26 in osteoclastogenesis in RA.

**Methods:**

We analyzed levels of IL-20 receptor subunit A (IL-20RA), CD55, and receptor activator of nuclear factor kappaB (NF-κB) ligand (RANKL) in RA fibroblast-like synoviocytes (FLSs) using confocal microscopy. Recombinant human IL-26-induced *RANKL* expression in RA-FLSs was examined using real-time polymerase chain reaction (PCR) and enzyme-linked immunosorbent assay (ELISA). Human peripheral blood monocytes were cultured with macrophage colony-stimulating factor (M-CSF) and IL-26, after which osteoclastogenesis was evaluated by counting the number of tartrate-resistant acid phosphatase-positive multinucleated cells. Additionally, osteoclastogenesis was evaluated by monocytes co-cultured with IL-26-prestimulated FLSs.

**Results:**

The expression of *IL-20RA* in RA-FLSs was higher than that in osteoarthritis-FLSs. Additionally, in IL-26-pretreated RA-FLSs, the expression of *IL-20RA* (but not *IL-10 receptor subunit B*) and *RANKL* increased in a dose-dependent manner, with IL-26-induced *RANKL* expression reduced by *IL-20RA* knockdown. Moreover, IL-26-induced *RANKL* expression was significantly downregulated by inhibition of signal transducer and activator of transcription 1, mitogen-activated protein kinase, and NF-κB signaling. Furthermore, IL-26 promoted osteoclast differentiation from peripheral blood monocytes in the presence of low dose of RANKL, with IL-26 exerting an additive effect. Furthermore, co-culture of IL-26-pretreated RA-FLSs with peripheral blood monocytes also increased osteoclast differentiation in the absence of addition of RANKL.

**Conclusions:**

IL-26 regulated osteoclastogenesis in RA through increased *RANKL* expression in FLSs and direct stimulation of osteoclast differentiation. These results suggest the IL-26/IL-20RA/RANKL axis as a potential therapeutic target for addressing RA-related joint damage.

## Background

Rheumatoid arthritis (RA) is a chronic inflammatory disease of autoimmune nature marked by synovial inflammation and subsequent structural damage of cartilage and subchondral bone [1]. The pathogenic hallmark of RA includes an expanded synovial membrane due to increased activation of synoviocytes accompanied by infiltration of innate and adaptive immune cells into the synovial sublining. This inflamed and proliferative synovial membrane, termed “pannus,” invades the periarticular bone at the cartilage–bone junction, leading to joint damage. At the side of the pannus, osteoclast activation mainly mediates the destruction of cartilage and subchondral bone [2, 3].

After the discovery of the type 17 T helper cell (Th17) subset, numerous studies have reported that Th17 cells play an important role in RA pathogenesis. Th17 cells are a distinct lineage of CD4^+^ Th cells characterized by the production of a variety of proinflammatory cytokines, including interleukin (IL)-17A, IL-17F, IL-6, IL-21, IL-22, IL-26, tumor necrosis factor (TNF)-α, and granulocyte-macrophage colony-stimulating factor [4]. Considerable evidence from both animal models and humans shows that IL-17 is involved not only in inflammatory cascades but also in joint damage through its activation of osteoclast differentiation (i.e., osteoclastogenesis) [4–6].

IL-26 is a 171-amino acid protein and a member of the Th17 cytokine family. Th1, Th17, and natural killer (NK) cells represent cellular sources of IL-26, with recent studies showing that IL-26 is also produced in various other cell types, including alveolar macrophages [7], fibroblast-like synoviocytes (FLSs), and macrophage-like synoviocytes in patients with RA [8] and myofibroblasts in patients with spondyloarthritis [9]. IL-26 binds to a heterodimeric receptor complex comprising IL-20 receptor subunit A (IL-20RA) and IL-10 receptor subunit B (IL10-RB) chains [10] to activate Janus tyrosine kinase (JAK)1/signal transducer and activator of transcription (STAT)1 and STAT3 pathways [11]. Although the IL-10RB monomer is broadly expressed on most cell types, the IL-20RA monomer is sparsely expressed on epithelial cells, keratinocytes, and myeloid cells. IL-22 binds receptor complexes containing IL-10RB/IL-22RA1, whereas IL-19, IL-20, and IL-24 bind the heterodimer receptor complex comprising IL-20RA/IL20RB [12].

The inflammatory cascade in the RA synovium is modulated by a variety of cytokine and chemokine networks; however, the roles of IL-26 in RA pathogenesis are poorly defined. A previous study reported that IL-26 mainly expressed by synovial cells induces the production of proinflammatory cytokines (IL-1-β, IL-6, and TNF-α) by myeloid cells and promotes Th17 generation from non-Th17-committed CD4^+^ memory T cells [8]. Receptor activator of nuclear factor-kappaB (NF-κB) ligand (RANKL), which is expressed by T cells, synovial fibroblasts, and stromal cells, mainly stimulates osteoclastogensis and bone resorption by binding to its receptor (RANK) on osteoclast progenitors. Cytokines, such as IL-1β, IL-6, IL-17, and TNF-α, promote RANKL expression in the RA synovium, leading to increased osteoclastogenesis; however, contrary to expectations, a recent study demonstrated that IL-26 inhibited osteoclastogenesis by downregulation of NF-κB activation and nuclear translocation of nuclear factor of activated T cells, cytoplasmic 1 (NFATc1) in RAW264.7 cells, a murine macrophage cell line [13]. Although mice and rats harbor endogenous IL-20RA and IL-10RB chains, the *Il-26* gene is absent in the murine genome [11]; therefore, the cellular functions of IL-26 in osteoclastogenesis associated with murine cell lines could differ from that in a human RA model. Therefore, the role of IL-26 in osteoclastogenesis in RA needs to be clarified in order to understand its role in RA pathogenesis.

In this study, we investigated the effect of IL-26 on RANKL production in FLSs and osteoclast differentiation from peripheral blood monocytes and also examined IL-26-mediated signaling pathways associated with induction of RA-related osteoclastogenesis.

## Methods

### Patients

Synovial tissues were isolated from eight RA patients (mean age 63.4 ± 4.6 years; range 38–76 years) and five osteoarthritis (OA) patients (mean age 56.6 ± 4.7 years; range 32–70 years) undergoing total knee-replacement surgery. Informed consent was obtained from all patients, and the experimental protocol was approved by the Institutional Review Board for Human Research, Konkuk University Hospital (KUH1010186).

### FLS isolation

FLSs were isolated by enzymatic digestion of synovial tissues obtained from RA and OA patients undergoing total knee-replacement surgery, as described previously [14]. To establish cell lines, synovial tissues were minced into 2- to 3-mm pieces and treated for 4 h with 4 mg/mL of type 1 collagenase (Worthington Biochemicals, Freehold, NJ, USA) in Dulbecco’s modified Eagle’s medium (DMEM) at 37 °C and 5% CO_2_. Dissociated cells were centrifuged at 500*g* and resuspended in DMEM supplemented with 10% fetal calf serum, 2 mM l-glutamine, 100 U/ml penicillin, and 100 μg/mL streptomycin. Suspended cells were plated in 75-cm^2^ culture flasks and were cultured at 37 °C and 5% CO_2_. Medium was replaced every 3 days, and once the primary culture reached confluence, cells were split weekly. Cells at passages five to eight contained a homogeneous population of FLSs.

### Reagents

Recombinant IL-26, RANKL, and macrophage colony-stimulating factor (M-CSF) were purchased from R&D Systems (Minneapolis, MN, USA). SR11302 [activator protein 1 (AP-1) inhibitor], fludarabine (a STAT1 inhibitor), and parthenolide (an NF-κB inhibitor) were obtained from Sigma-Aldrich (St. Louis, MO, USA). LY294002 [a phosphoinositide 3-kinase (PI3K) inhibitor], SB203580 [a p38 mitogen-activated protein kinase (MAPK) inhibitor], PD98059 [an extracellular signal-regulated kinase (ERK) inhibitor], and AG490 [a Janus kinase (JAK)2 inhibitor] were obtained from Calbiochem (Schwalbach, Germany).

### Confocal microscopy

To measure changes in protein expression, synovial fibroblasts were fixed in 4% formaldehyde–phosphate-buffered saline (PBS) for 15 min at 37 °C, permeabilized, and incubated for 15 min with 0.5% Triton X–100 (v/v) (Sigma-Aldrich). Fixed cells were washed and incubated with primary antibodies against RANKL (Santa Cruz Biotechnology, Dallas, TX, USA), IL-20RA (Santa Cruz Biotechnology), and CD55 (Bio-Rad, Hercules, CA, USA), a typical marker of synovial fibroblasts [15], at 4 °C overnight.

Cells were then washed and incubated with secondary anti-mouse antibodies conjugated to fluorescein isothiocyanate (Santa Cruz Biotechnology), anti-rabbit phycoerythrin (Southern Biotech, Birmingham, AL), and anti-mouse IgG2a–peridinin-chlorophyll protein Cy5.5 (Southern Biotech, Birmingham, AL, USA). The stained sections were visualized under a Zeiss microscope (LSM 510 Meta; Carl Zeiss, Oberkochen, Germany) at × 200 and × 400 magnifications.

### Real-time polymerase chain reaction (PCR)

FLSs were stimulated with various concentrations of IL-26 (1, 10, 20, 50, and 100 ng/mL). For RANKL signal-pathway analysis, FLSs were incubated in the presence or absence of SR11302 (1 μM), fludarabine (0.5 μM), parthenolide (10 μM), Ly294002 (20 μM), SB203580 (10 nM), PD98059 (20 μM), or AG490 (50 μM) for 1 h prior to the addition of IL-26. After incubation for 72 h, mRNA was extracted using RNAzol B (Biotex Laboratories, Houston, TX, USA) according to manufacturer instructions. Reverse transcription of 2 μg of total mRNA was performed at 42 °C using the Superscript reverse transcription system (Takara, Shiga, Japan). PCR was performed in a 20-μL final volume in capillary tubes in a LightCycler instrument (Roche Diagnostic, Mannheim, Germany), with the reaction mixture containing 2 μL of LightCycler FastStart DNA MasterMix for SYBR Green I (Roche Diagnostic), 0.5 μM of each primer, 4 mM MgCl_2_, and 2 μL of template DNA. All capillaries were sealed, centrifuged at 500*g* for 5 s, and amplified in a LightCycler instrument (Roche Diagnostic) using the following thermal conditions: polymerase activation at 95 °C for 10 min, followed by 45 cycles of 10 s at 95 °C, 10 s at 60 °C (β-actin), 58 °C (IL-20RA), 57 °C (IL-10RB) or 59 °C (RANKL), and 10 s at 72 °C. The temperature transition rate was 20 °C/s for all steps. The PCR product was measured during the 72 °C extension step by detection of fluorescence associated with the binding of SYBR Green I to the product. Fluorescence curves were analyzed with LightCycler software (v.3.0; Roche Diagnostics). The LightCycler was used to quantify *RANKL* mRNA by calculating these levels relative an endogenously expressed housekeeping gene (*β-actin*). Melting curve analysis was performed immediately after the amplification protocol under the following conditions: 0 s (hold time) at 95 °C, 15 s at 71 °C, and 0 s (hold time) at 95 °C. The rate of temperature change was 20 °C/s, except for 0.1 °C/s used in the final step. The generated melting peak represented the amount of specific amplified product. The crossing point (*C*_p_) was defined as the maximum of the second derivative from the fluorescence curve. Negative controls contained all of the elements of the reaction mixture, except for template DNA. All samples were processed in duplicate.

### Reverse transcription (RT)-PCR

FLSs were incubated with various concentrations of IL-26, and after incubation for 72 h, mRNA was extracted using RNAzol B (Biotex Laboratories, Houston, TX, USA) according to manufacturer instructions. Reverse transcription of 2 μg of total mRNA was performed at 42 °C using the Superscript reverse transcription system (Takara). PCR amplification of cDNA aliquots was performed by adding 2.5 mM dNTPs, 2.5 U Taq DNA polymerase (Takara), and 0.25 μM of sense and antisense primers to PCR buffer [1.5 mM MgCl_2_, 50 mM KCl, and 10 mM Tris-HCl (pH 8.3)] at a total volume of 25 μL. The sense and antisense primers for each molecule (5′ → 3′) are listed in Additional file 1: Table S1. Reactions were processed in a DNA thermal cycler (PerkinElmer Cetus, Wellesley, MA, USA), and PCR products were electrophoresed on a 2% agarose gel and stained with ethidium bromide. Results are expressed as the ratio of *RANKL* (forward primer, 5′-ACC-AGC-ATC-AAA-ATC-CCA-AG-3′; and reverse primer, 5′-CCC-CAA-AGT-ATG-TTG-CAT-CC-3′) to *β-actin* (forward primer, 5′-GGA-CTT-CGA-GCA-AGA-GAT-GG-3′; and reverse primer, 5′-TGT-GTT-GGG-GTA-CAG-GTC-TTT-G-3′) PCR products.

### Enzyme-linked immunosorbent assay (ELISA)

Briefly, a 96-well plate (Nunc; Sigma-Aldrich) was coated with 4 μg/mL of monoclonal antibody against soluble (s) RANKL (R & D Systems) at 4 °C overnight. After blocking with PBS/1% bovine serum albumin (BSA)/0.05% Tween-20 for 2 h at room temperature (22–25 °C), test samples and the standard recombinant sRANKL (R & D Systems) were added to the 96-well plate and incubated at room temperature for 2 h. Plates were washed four times with PBS/Tween-20 and incubated with 500 ng/mL of biotinylated mouse monoclonal antibody against sRANKL (R & D Systems) for 2 h at room temperature. After washing, a streptavidin–alkaline phosphate–horseradish-peroxidase (HRP) conjugate (Sigma-Aldrich) was added and incubated for 2 h, followed by washing and subsequent incubation with 1 mg/mL *p*-nitrophenyl phosphate (Sigma-Aldrich) dissolved in diethanolamine (Sigma-Aldrich) to develop the color reaction. The reaction was stopped by addition of 1 M NaOH, and the optical density of each well was read at 405 nm. The lower limit of sRANKL detection was 10 pg/mL, and recombinant human sRANKL diluted in culture medium was used as a calibration standard (range 10–2000 pg/mL). A standard curve was generated by plotting the optical density against the log of the concentration of recombinant cytokines and subsequently used to determine sRANKL concentrations in test samples.

### Western blot analysis

FLSs were incubated with IL-26, and after a 1-h incubation, whole-cell lysates were prepared from ~ 2 × 10^5^ cells by homogenization in lysis buffer and centrifugation at 14,000 rpm for 15 min. Protein concentration in the supernatant was determined using a Bradford assay kit (Bio-Rad, Hercules, CA, USA). Protein samples were separated by 10% sodium dodecyl sulfate polyacrylamide electrophoresis and transferred to a nitrocellulose membrane (Amersham Pharmacia Biotech, Uppsala, Sweden). For western hybridization, the membrane was preincubated with 0.5% skim milk in TBST (Tris-buffered saline with 0.1% Tween-20) at room temperature for 2 h. Primary antibodies against phospho-Src homology region 2 domain-containing phosphatase-1 (SHP-1), SHP-1, phospho-Syk, Syk, phospho-STAT1, STAT1, phospho-STAT3(Y705), phospho-STAT3(S727), STAT3, phospho-ERK, ERK, phospho-JNK, JNK, ho-p38, p38, phospho-inhibitor of kappaBα (IκBα), IκBα, phospho-c-Jun, or c-Jun (1:1000; Cell Signaling Technology, Danvers, MA, USA). The anti-β-actin antibody (1:3000; Sigma-Aldrich) was used as an internal control following 1:1000 dilution in 5% BSA–TBST prior to overnight incubation at 4 °C. The membrane was washed four times with TBST, and HRP-conjugated secondary antibody was added and incubated for 1 h at room temperature. After TBST washing, hybridized bands were detected using an enhanced chemiluminescence detection kit and Hyperfilm-ECL reagents (Amersham Pharmacia Biotech).

### Oligonucleotide magnetofection

*IL-20RA* short-hairpin (sh) RNA plasmids (h) were transfected using magnetofection, as described previously [16, 17], with assays after performed 24 h. *IL-20RA*-shRNA and control plasmids were purchased from Santa Cruz Biotechnology.

### Monocyte isolation and osteoclast differentiation

Monocytes were isolated from peripheral blood mononuclear cells (PBMCs) as follows: healthy donor PBMCs were separated from whole blood by Ficoll gradients (Ficoll-Paque Plus; GE Healthcare, Uppsala, Sweden), followed by incubation in a 48-well plate at 37 °C in a humidified atmosphere containing 5% CO_2_. After 2 h, non-adherent cells were removed by repeated washes with complete Roswell Park Memorial Institute medium to reveal adherent primary monocytes. Non-adherent cells were removed, and adherent cells were washed with sterile PBS, harvested with a rubber policeman, and stained with the monocyte-specific anti-CD14 monoclonal antibody to assess preparation purity. Our results showed that 90% of the isolated cells were CD14^+^ monocytes. The medium was replenished every 2 to 3 days, and after 14 days, the cells were stained for tartrate-resistant acid phosphatase (TRAP) according to manufacturer instructions (Sigma-Aldrich). Wells were visualized by light microscopy, and TRAP^+^ cells with three or more nuclei were counted as osteoclasts.

### Bone-resorption assay

We performed an in vitro resorption pit assay using a bone-resorption assay kit (Cosmo Bio Co., Ltd., Tokyo, Japan). Monocytes were cultured on a bone-coating plate with M-CSF in the presence or absence of various concentrations of IL-26 (1, 50, and 100 ng/mL) for 14 days. The cells were removed from the bone-coating plate by wiping the surface, and the numbers of pits formed by bone resorption on the plate were counted.

### Statistical analysis

Data are expressed as the mean ± standard error of the mean (SEM). Statistical analysis was performed using one-way analysis of variance, followed by Dunnet’s multiple comparison test for comparison of more than two experimental groups. A *P* < 0.05 was considered statistically significant.

## Results

### IL-20RA and RANKL levels in RA-FLSs

**Fig. 1:**
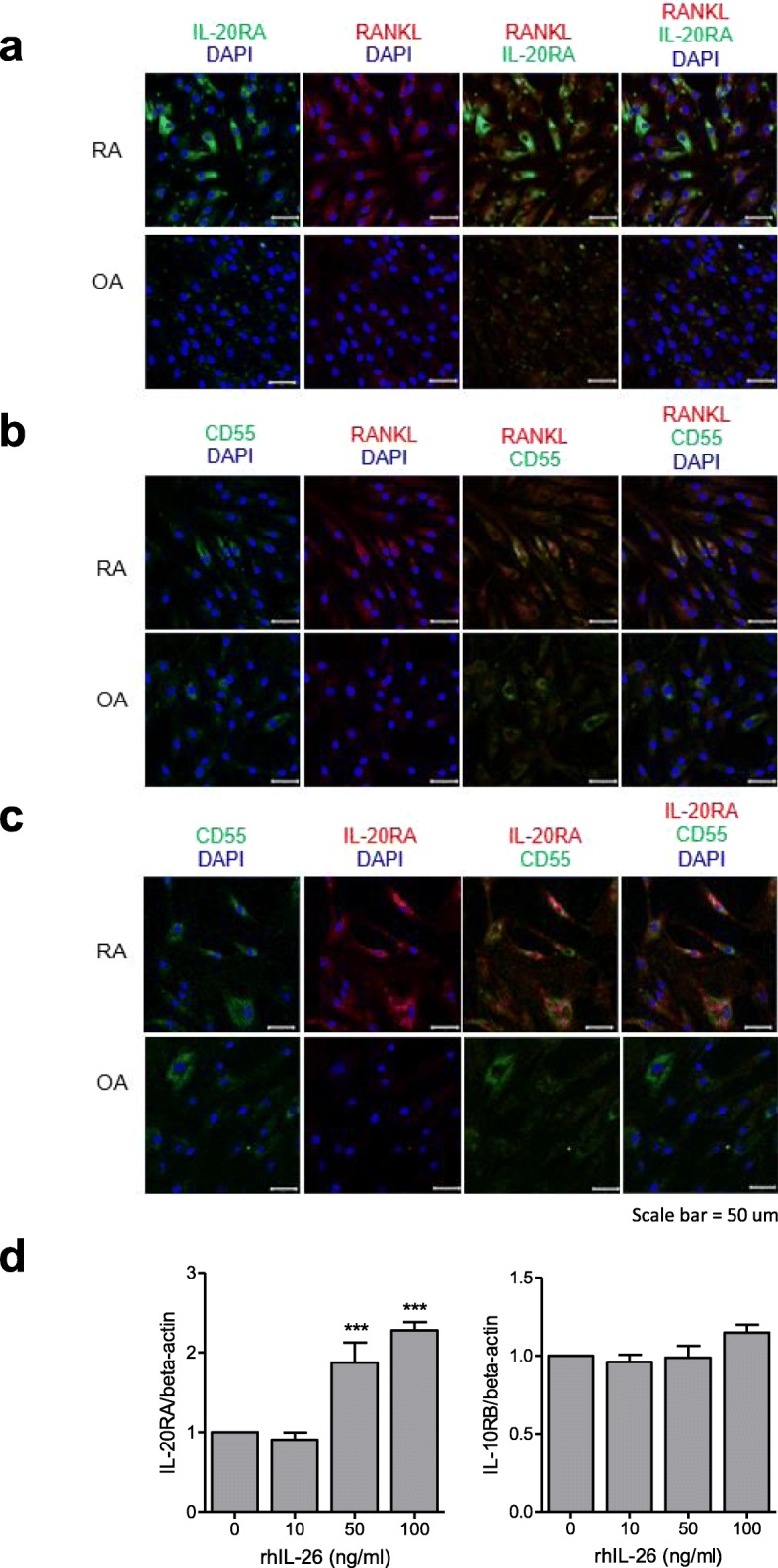
IL-20RA and RANKL in FLS from RA patients. **a** RA- and OA-FLSs were simultaneously labeled with anti-IL-20RA (green) and anti-RANKL (red) antibodies and then photographed under appropriate filters (*n* = 3). **b** RA and OA-FLSs were simultaneously labeled with anti-CD55 (green) and anti-RANKL (red) antibodies and then photographed under appropriate filters (*n* = 3). **c** RA and OA-FLSs were simultaneously labeled with anti-CD55 (green) and anti-IL-20RA (red) antibodies and then photographed under appropriate filters (*n* = 3). The merged image shows co-localization of the two markers (yellow). Sections were counterstained with 4′,6-diamidino-2-phenylindole. Images are representative of three independent experiments (original magnification × 200). **d** RA-FLSs were cultured with IL-26 at various concentrations, and *IL-20RA* and *IL-10 receptor subunit B* mRNA levels were determined by real-time polymerase chain reaction (*n* = 3). Results are expressed as the SEM of three independent experiments. ***P < 0.005. IL-20RA interleukin (IL)-20 receptor subunit A, RANKL receptor activator of nuclear factor-kappaB ligand, FLSs fibroblast-like synoviocytes, RA rheumatoid arthritis, OA osteoarthritis

To investigate the expression of IL-20RA and RANKL in RA and OA-FLS, we investigated their levels by confocal microscopy with multiple-fluorescence staining. We found that IL-20RA, RANKL, and CD55 were abundantly expressed in RA-FLSs but negligibly expressed in OA-FLSs and that dual-immunofluorescent labeling of IL-20RA and RANKL revealed consistent overlaps between RANKL, IL-20RA, and CD55 (Fig. 1a–c). These results indicated that CD55^+^ RA-FLS expressed both IL-20RA and RANKL. We then cultured RA-FLSs with IL-26 at various concentrations and determined *IL-20RA* and *IL-10RB* mRNA level by real-time PCR. Stimulation with IL-26 increased *IL-20RA* mRNA levels in a dose-dependent manner, with 100 ng/mL IL-26 producing the maximal effect; however, no IL-26-mediated alteration of *IL-10RB* levels was observed in RA-FLSs (Fig. 1d). In OA-FLSs, IL-26 did not increase the expression of *IL-20RA* or *IL-10RB*, suggesting that IL-26-mediated alteration of IL-20RA is specific for RA-FLSs (Additional file 1: Figure S1).

### IL-26 stimulates *RANKL* expression in RA-FLS

**Fig. 2:**
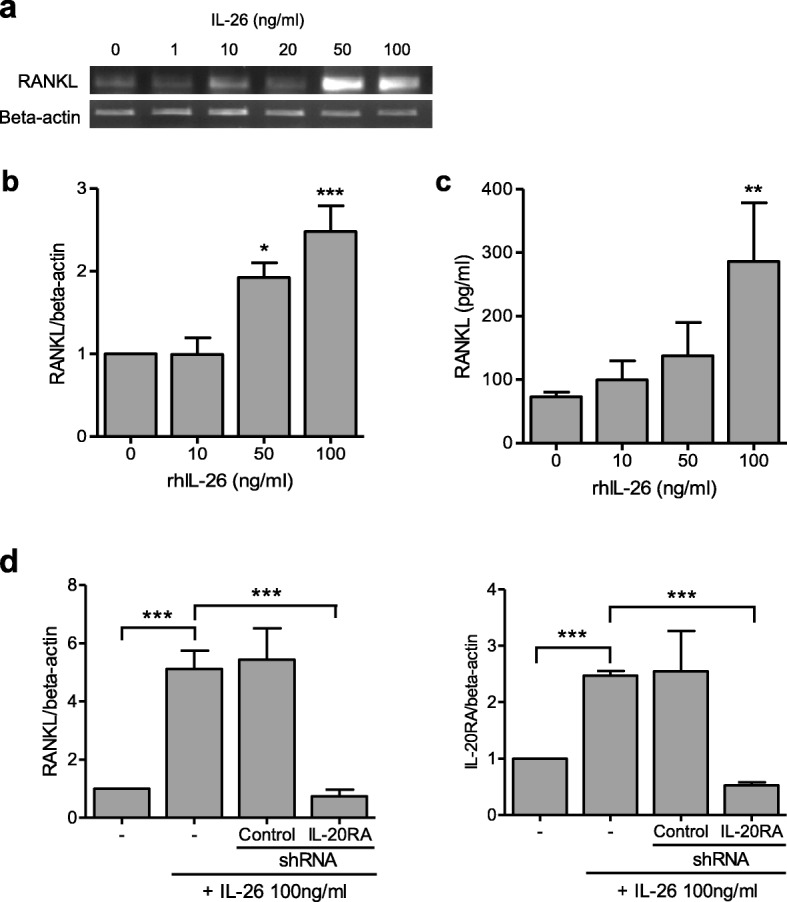
IL-26 induces *RANKL* expression in RA-FLSs. Following culture of RA-FLSs (*n* = 3) with 0–100 ng/mL of recombinant human (rh)IL-26 for 72 h, RANKL mRNA levels were determined by **a** reverse transcription PCR and **b** real-time PCR. Data were normalized to β-actin levels and presented as relative expression units. The image is representative of three experiments. **c** RA-FLSs were cultured with rhIL-26 for 72 h, and RANKL concentration in the culture media was measured by sandwich enzyme-linked immunosorbent assay. **d** RA-FLSs were cultured with 100 ng/mL of rhIL-26 and *IL-20RA* short-hairpin (sh) RNA or control shRNA for 72 h, and *RANKL* and *IL-20 receptor subunit A* mRNA levels were determined by real-time PCR. Target gene expression was normalized against *β-actin* expression. Data represent the mean ± standard error of the mean of three independent experiments. **P < 0.01; ***P < 0.005. IL interleukin, RANKL receptor activator of nuclear factor-kappaB ligand, RA rheumatoid arthritis, FLSs fibroblast-like synoviocytes, PCR polymerase chain reaction

RA-FLSs were cultured with IL-26 at various concentrations, and RANKL mRNA and protein levels were measured using RT-PCR, real-time PCR, and ELISA. Stimulation with IL-26 increased RANKL mRNA (Fig. 2a, b) and protein (Fig. 2c) levels in a dose-dependent manner; however, we found that stimulation of RA-FLSs with IL-26 did not change IL-1β, IL-6, and TNF-α levels in the cultured media (Additional file 1: Figure S2). We then transfected RA-FLSs with *IL-20RA* shRNA, finding that *IL-20RA* knockdown blocked IL-26-induced *RANKL* and *IL-20RA* expression (Fig. 2d).

### Intracellular signaling pathways associated with IL-26-induced *RANKL* expression

**Fig. 3:**
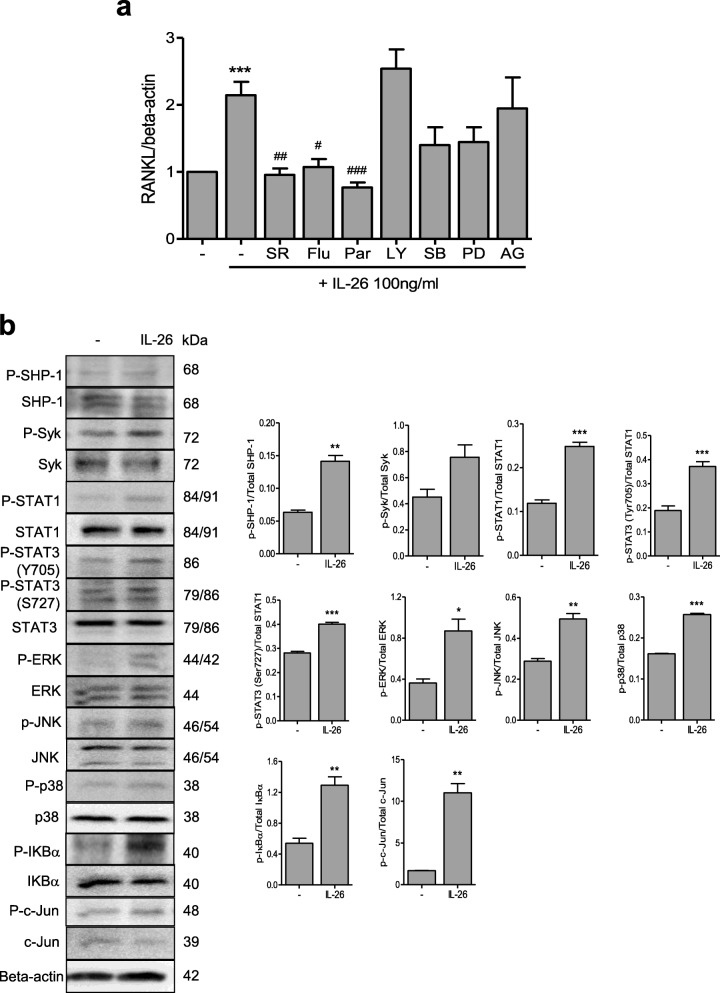
Downstream signaling pathways mediating IL-26-induced *RANKL* expression in RA-FLSs. **a** RA-FLSs (*n* = 3) were pretreated with SR11302 (AP-1 inhibitor) (1 μM), fludarabine (STAT1 inhibitor) (0.5 μM), parthenolide (NF-κB inhibitor) (10 μM), Ly294002 (PI3K inhibitor) (20 μM), SB203580 (MAPK inhibitor) (10 nM), PD98059 (ERK inhibitor) (20 μM), or AG490 (JAK2 inhibitor) (50 μM) for 1 h, followed by culture with 100 ng/mL of IL-26 for 72 h. *RANKL* mRNA level was quantified using quantitative real-time polymerase chain reaction and normalized against *β-actin* expression. Data were expressed as relative *RANKL/β-actin* level. **b** RA-FLSs (*n* = 3) were cultured with 100 ng/mL IL-26, and phosphorylation of signaling-related molecules was assessed in cell lysates using western blot. Data were expressed relative to β-actin level and represent the mean ± standard error of the mean of three independent experiments. ***P < 0.005 vs. control; ^#^*P* < 0.05; ^##^*P* < 0.01; ^###^*P* < 0.005 vs. IL-26. IL interleukin, RANKL receptor activator of nuclear factor-kappaB ligand, RA rheumatoid arthritis, FLSs fibroblast-like synoviocytes

To identify the intracellular signaling pathways mediating IL-26-induced *RANKL* expression, RA-FLSs were preincubated with signaling inhibitors for 1 h and then cultured with IL-26 for 1 h. We found that *RANKL* mRNA levels decreased significantly after inhibition of AP-1-, STAT1-, and NF-κB-related signaling (Fig. 3a), with no cytotoxic effects of the inhibitors observed on FLSs under the experimental concentrations. Additionally, western blot revealed that IL-26 induced the phosphorylation of SHP-1, STAT1, STAT3(T705), STAT3(S727), ERK, JNK, p38 MAPK, IκBα, and c-Jun in FLSs (*P* < 0.05 for ERK; *P* < 0.01 for SHP-1, JNK, IκBα, and c-Jun; *P* < 0.001 for STAT1, STAT3(T705), STAT3(S727), and p38 MAPK) (Fig. 3b).

### IL-26 induces osteoclast differentiation from PBMCs

To investigate the expression of the IL-26 receptor in osteoclasts, osteoclasts were cultured with IL-26 at various concentrations, and *IL-20RA* and *IL-10RB* mRNA levels were determined by real-time PCR. Stimulation with IL-26 increased *IL-20RA* mRNA levels in a dose-dependent manner, whereas IL-26-mediated alteration of *IL-10RB* levels was not observed in osteoclasts (Additional file 1: Figure S3).

**Fig. 4:**
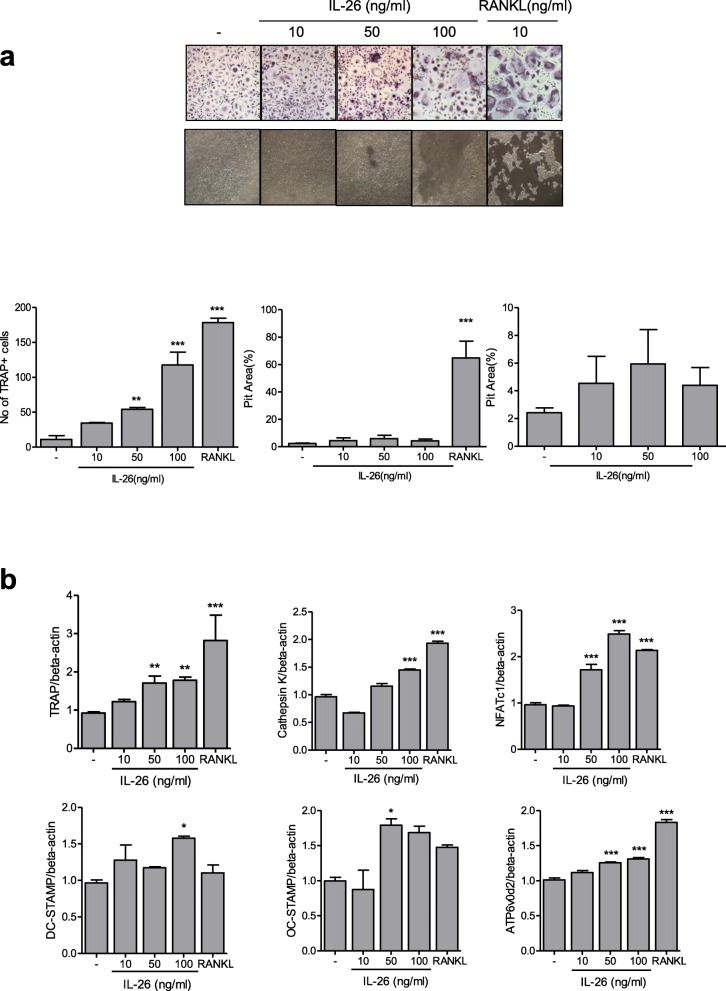
IL-26 promotes the differentiation of peripheral blood monocytes into osteoclasts. **a** Human peripheral blood CD14^+^ monocytes (*n* = 3) were isolated and cultured with 0 (-), 10, 50, and 100 ng/mL recombinant human (rh)IL-26 or 10 ng/mL RANKL in the presence of macrophage colony-stimulating factor (25 ng/mL). Osteoclast differentiation was determined by counting tartrate-resistant acid phosphatase (TRAP)^+^ multinucleated cells with at least three nuclei. A bone-resorption assay demonstrating IL-26-induced bone-resorbing activity. Images are representative of three independent experiments. **b** The expression of osteoclast markers was determined using real-time polymerase chain reaction, with mRNA levels normalized against *β-actin* expression (*n* = 3). Data are presented as means ± standard error of the mean of three separate experiments. *P < 0.05; **P < 0.01; ***P < 0.005. IL interleukin, CD cluster designation, RANKL receptor activator of nuclear factor-kappaB ligand

Then, to identify the effect of IL-26 on the induction of osteoclastogenesis, isolated CD14^+^ monocytes from the peripheral blood were cultured with IL-26 and M-CSF in the absence of addition of RANKL. After a 14-day culture, TRAP^+^ multinucleated osteoclasts were differentiated from the monocytes in the IL-26 and M-CSF culture system in the absence of addition of RANKL, although the number and size of the differentiated osteoclasts were lower than those in the traditional culture system that included RANKL. Additionally, a bone-resorption assay showed that IL-26 induced bone-resorbing activity, but the effect was not significant. The number of pits formed by IL-26-induced bone resorption was lower than that formed by RANKL (Fig. 4a).

In the absence of addition of RANKL, multiple TRAP^+^ multinucleated osteoclasts were observed in the presence of M-CSF only (Fig. 4a). Therefore, we evaluated RANKL concentration in culture supernatants. RANKL was present at low concentrations, which did not increase following stimulation with M-CSF alone, suggesting that IL-26 induced the effect at low doses of RANKL **(**Additional file 1: Figure S4).

**Fig. 5:**
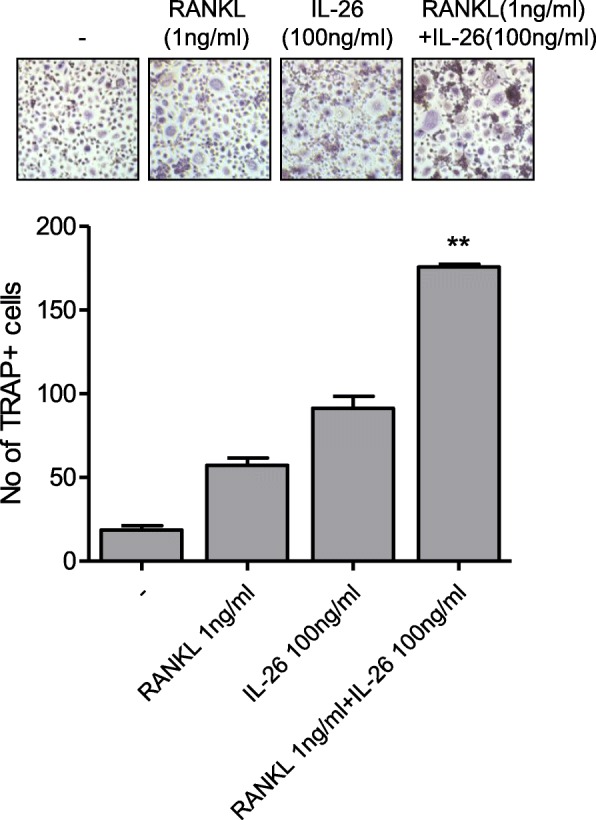
IL-26 promotes monocyte differentiation into osteoclasts with *RANKL*. Peripheral blood CD14^+^ monocytes (*n* = 3) were cultured with 25 ng/mL macrophage colony-stimulating factor (M-CSF) only (-) or with M-CSF (25 ng/mL) plus 1 ng/mL RANKL and/or 100 ng/mL IL-26. Images are representative of three independent experiments. **P < 0.01. IL interleukin, RANKL receptor activator of nuclear factor-kappaB ligand, CD cluster designation

Moreover, levels of osteoclast markers, including TRAP, cathepsin K, NFATc1, dendritic cell (DC)-specific seven transmembrane protein (STAMP), osteoclast (OC)-specific STAMP, and V0-complex subunit of vacuolar ATPase (ATP6Vod2), were significantly increased by IL-26 stimulation (*P* < 0.05) (Fig. 4b). These results indicated an additive effect on osteoclast differentiation upon co-culture of PBMCs with IL-26 and RANKL (Fig. 5).

**Fig. 6:**
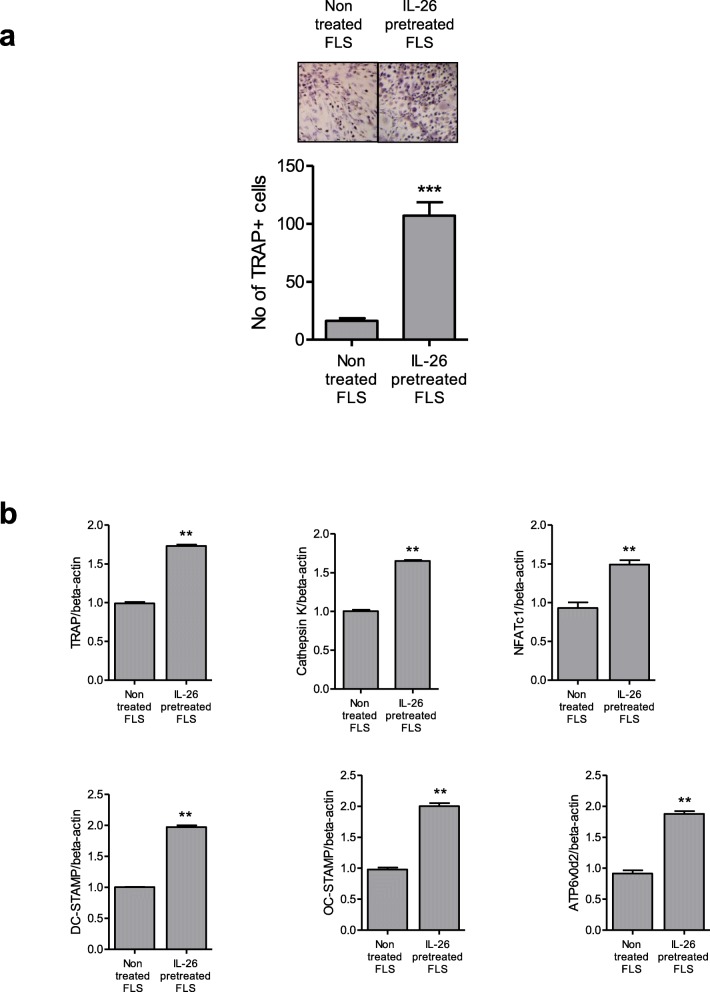
IL-26-pretreated RA-FLSs induce osteoclastogenesis from peripheral blood monocytes. **a** RA-FLSs (*n* = 3) were preincubated with 100 ng/mL IL-26 for 72 h and then co-cultured with CD14^+^ monocytes from peripheral blood in the presence of macrophage colony-stimulating factor. After a 21-day culture, tartrate-resistant acid phosphatase (TRAP)^+^ multinucleated cells were counted. The image is representative of three independent experiments. **b** mRNA levels of osteoclast markers from differentiated osteoclasts according to real-time polymerase chain reaction (*n* = 3). Data were normalized against *β-actin* expression and represent the mean ± standard error of the mean of three independent experiments. **P < 0.01; ***P < 0.005. IL interleukin, RA rheumatoid arthritis, FLSs fibroblast-like synoviocytes, CD cluster designation

To determine the indirect effects of IL-26 on osteoclastogenesis, RA-FLSs were pretreated with IL-26, followed by their addition to a culture with PBMCs and M-CSF. Upon co-culture of isolated monocytes with IL-26-pretreated RA-FLSs in the absence of RANKL, we observed differentiation of TRAP^+^ multinucleated cells (Fig. 6a) accompanied by significant increases in the osteoclast markers TRAP, cathepsin K, NFATc1, DC-STAMP, OC-STAMP, and ATP6Vod2 (Fig. 6b; **P < 0.01, ***P < 0.001).

## Discussion

In this study, we found that IL-26, a newly identified IL-17 family cytokine, induced osteoclastogenesis in two ways. First, IL-26 in the presence of M-CSF enhanced osteoclast differentiation from PBMCs at low concentration of RANKL. Second, we found that co-culture of IL-26-prestimulated RA-FLSs and PBMCs in the absence of addition of RANKL increased osteoclast differentiation.

Most effector cytokines associated with Th17 cells are involved in osteoclast maturation and activation via induction of the RANKL-RANK-osteoprotegerin system [4]; therefore, we hypothesized that IL-26 plays a role in driving osteoclastogensis. However, a recent study observed IL-26-specific inhibitory activity on osteoclastogenesis through downregulation of RANKL-induced NF-kB and NFATc1 levels in murine RAW264.7 cells [13]. Although the *IL-26* gene is conserved in most vertebrate species, it is curiously absent from mice, which hinders the characterization of this cytokine in vivo [18]. To confirm the pathogenic role of IL-26 in RA, we investigated its function in osteoclastogenesis using human primary cells (peripheral blood monocytes, osteoclasts, and FLSs) and RA synovial tissues. We designed a co-culture system involving monocytes and IL-26-prestimulated FLSs in order to mimic the RA synovial setting, given that FLSs are key promoters of bone erosion based on their capacity to express RANKL [19]. Our results suggested that IL-26 promoted osteoclastogenesis in RA via two pathways: (1) direct effects associated with induced differentiation of osteoclast precursors and (2) indirect effects through increased *RANKL* expression in FLSs. However, a bone-resorption assay showed that IL-26 did not have functional activity as much as RANKL. Therefore, our study suggests that indirect effects via increased RANKL expression in FLSs might play more important role in osteoclastogenesis in RA than direct enhancing differentiation of osteoclast precursors.

Our findings demonstrated increased basal expression of *IL-20RA* and co-expression with *RANKL* in RA-FLSs, with IL-26 promoting *IL-20RA* expression but not *IL-10RB* in RA-FLSs. However, OA-FLSs showed no IL-26-mediated change in *IL-20RA* expression, suggesting that IL-26 plays a role in the pathogenesis of RA but not of OA. Additionally, IL-26-induced RANKL expression in RA-FLSs was significantly decreased by shRNA-mediated knockdown of IL-20RA. These results suggested that an IL-26-mediated signaling pathway in RA-FLSs is dependent upon IL-20RA. On the other hand, a previous report showed that IL-26 induces IL-10RB-related (but not IL-20RA-related) signaling in monocytes, with IL-26-induced monocyte activation associated with IL-10RB phosphorylation and subsequently decreased in the presence of a neutralizing anti-IL-10RB monoclonal antibody [8]. It is possible that the main regulatory ligands of IL-20RA and IL-10RB might differ depending on cell type, as other reports described IL-26-receptor-independent effects on human B cells [20], various epithelial cell types, and primary human foreskin fibroblasts [21]. Additionally, the highly cationic and amphipathic nature of IL-26 could facilitate binding to various molecules presented on cell surfaces, suggesting the possible existence of an unknown IL-26-specific receptor other than IL-20RA/IL-10RB [22]. Therefore, characterization of IL-26-receptor expression by target cell types is needed.

Although a previous study reported that IL-26 increased the secretion of IL-1β, IL-6, and TNF-α by myeloid cells in RA synovial fluid [8], we did not observe upregulated levels of these cytokines in IL-26-stimulated RA-FLSs, suggesting that IL-26 did not mediate the production of proinflammatory cytokines in RA-FLSs. Similar to the diversity of regulatory ligands for the IL-26 receptor, the effect of IL-26 on the production of proinflammatory cytokines could also be cell-type-dependent.

Intracellular signals associated with IL-26 involve the JAK-STAT pathway, which primarily activate STAT3 [10]. In the present study, our data indicated that IL-26 induced *RANKL* expression in RA-FLS via not only STAT1/3 but also the MAPK (ERK, p38 MAPK, and JNK) and NF-κB pathways. Moreover, the PI3K pathway, which regulates RA-FLS migration and invasion [19], was not activated by IL-26 stimulation. This agreed with the findings of other studies, including that IL-26 enhances the proliferation and tube formation of vascular endothelial cells via the Akt, ERK, and NF-κB pathways in a psoriasis-like murine model [22]. Additionally, in intestinal epithelial cells from patients with Crohn’s disease, IL-26 activates STAT1/3, ERK1/2, stress-activated protein kinase/JNK1/2, and Akt phosphorylation, leading to increased expression of proinflammatory cytokines [23].

The limitations of this study include the small sample size; therefore, confirmation of our findings using a larger sample is warranted. Additionally, we focused on the functional role of IL-26 in osteoclasts and RA-FLSs, whereas activated CD4^+^ T cells also play an important role in osteoclastogenesis through *RANKL* expression. Our study was limited to evaluation of the pathways associated with IL-26-induced osteoclastogenesis, including intracellular signaling pathways and blockage of the IL-20RA receptor on osteoclasts. Therefore, further studies are needed to reveal the effect of IL-26 on interactions between osteoclasts, FLSs, and T cells as well as downstream pathways related to IL-26-induced osteoclastogenesis.

## Conclusions

We demonstrated for the first time that IL-26 regulates osteoclastogenesis in RA through increased *RANKL* expression in FLSs and direct stimulation of osteoclast differentiation. Additionally, IL-26-induced *RANKL* expression in RA-FLSs occurred via IL-20RA and was mediated by the JAK-STAT, MAPK, and NF-κB pathways. Further evaluation of all the pathways associated with IL-26-induced osteoclastogenesis could contribute to elucidating the mechanisms of joint damage in RA.

These results suggest the IL-26-IL–20RA–RANKL axis as a potential therapeutic target for addressing joint damage in RA.

## Supplementary information

**Additional file 1 Figure S1.** Expression of IL-20RA and IL-10RB in FLSs from osteoarthritis OA patients. OA-FLSs were cultured with IL-26 at various concentrations, and the IL-20RA and IL-10RB mRNA levels were determined by real-time PCR. Results are presented as mean ± SEM (*n* = 3). **Figure S2.** Effect of IL-26 on proinflammatory cytokine levels in FLSs from RA patients. Following culture of RA synovial fibroblasts with rhIL-26 for 72 h, concentrations of TNF-α, IL-6, and IL-1β in the culture media were determined by sandwich ELISA. Results are presented as mean ± SEM (*n* = 3). **Figure S3.** Expression of IL-20RA and IL-10RB in osteoclasts. Osteoclasts were cultured with IL-26 at various concentrations, and IL-20RA and IL-10RB mRNA levels were determined by real-time PCR. Results are presented as mean ± SEM (n = 3). ***P < 0.005. **Figure S4.** RANKL concentration in culture media. RANKL concentration in the culture media without and with 25 ng/mL macrophage colony stimulating factor (M-CSF) was determined by sandwich ELISA. RANKL concentrations in the culture media were also evaluated following incubation with 0–100 ng/mL rhIL-26 for 72 h. The results are presented as mean ± SEM (n = 3). **Table S1.** Primer sequence (5′ → 3′).

## Data Availability

The datasets generated and/or analyzed in this study are available from the corresponding author upon reasonable request.

## Abbreviations

AP-1: Activator protein 1
ATP6Vod2: V0-complex subunit of vacuolar ATPase
BSA: Bovine serum albumin
DC: Dendritic cell
ELISA: Enzyme-linked immunosorbent assay
ERK: Extracellular signal-regulated kinase
FLS: Fibroblast-like synoviocyte
HRP: Horseradish peroxidase
IL: Interleukin
IL-10RB: IL-10 receptor subunit B
IL-20RA: IL-20 receptor subunit A
JAK: Janus kinase
JNK: c-Jun N-terminal kinase
MAPK: Mitogen-activated protein kinase
M-CSF: Macrophage colony-stimulating factor
NF-κB: Nuclear factor-kappaB
NFATc1: Nuclear factor of activated T cells, cytoplasmic 1
NK: Natural killer
OA: Osteoarthritis
OC: Osteoclast
PBMC: Peripheral blood mononuclear cells
PBS: Phosphate-buffered saline
PCR: Polymerase chain reaction
PI3K: Phosphoinositide 3-kinase
RA: Rheumatoid arthritis
RANKL: Receptor activator of NF-κB ligand
RT-PCR: Reverse transcription PCR
SHP-1: Src homology region 2 domain-containing phosphatase 1
shRNA: Short-hairpin RNA
sRANKL: Soluble RANKL
STAMP: Seven transmembrane protein
STAT: Signal transducer and activator of transcription
TBST: Tris-buffered saline with 0.1% Tween-20
Th: T helper
TNF: Tumor necrosis factor
TRAP: Tartrate-resistant acid phosphatase
