# Supplementary material for: Promotion of osteoclastogenesis by IL-26 in rheumatoid arthritis
Source: Arthritis Res Ther. 2019 Dec 12;21:283. doi: 10.1186/s13075-019-2070-0 (PMC6909469; doi:10.1186/s13075-019-2070-0)
Supplement: Supplementary file 1 — Additional file 1 Figure S1. Expression of IL-20RA and IL-10RB in FLSs from osteoarthritis OA patients. OA-FLSs were cultured with IL-26 at various concentrations, and the IL-20RA and IL-10RB mRNA levels were determined by real-time PCR. Results are presented as mean ± SEM (n = 3). Figure S2. Effect of IL-26 on proinflammatory cytokine levels in FLSs from RA patients. Following culture of RA synovial fibroblasts with rhIL-26 for 72 h, concentrations of TNF-α, IL-6, and IL-1β in the culture media were determined by sandwich ELISA. Results are presented as mean ± SEM (n = 3). Figure S3. Expression of IL-20RA and IL-10RB in osteoclasts. Osteoclasts were cultured with IL-26 at various concentrations, and IL-20RA and IL-10RB mRNA levels were determined by real-time PCR. Results are presented as mean ± SEM (n = 3). ***P < 0.005. Figure S4. RANKL concentration in culture media. RANKL concentration in the culture media without and with 25 ng/mL macrophage colony stimulating factor (M-CSF) was determined by sandwich ELISA. RANKL concentrations in the culture media were also evaluated following incubation with 0–100 ng/mL rhIL-26 for 72 h. The results are presented as mean ± SEM (n = 3). Table S1. Primer sequence (5′ → 3′). [file 13075_2019_2070_MOESM1_ESM.docx]

**Additional file 1**

**Table S1. Primer sequence (5′→3′)**

| primer |  | Sequence |
| --- | --- | --- |
| IL-20RA | forward | GGG TAT GCT TCG CAT TTG AT |
|  | reverse | GCA AAC TGA GCT CCT GCT CT |
| IL-10RB | forward | GCC TGT CTG TGA GCA AAC AA |
|  | reverse | AGG ATG GCC CAA AAA CTC TT |
| RANKL | forward | ACC AGC ATC AAA ATC CCA AG |
|  | reverse | CCC CAA AGT ATG TTG CATCC |
| TRAP | forward | GAC CAC CTT GGC AAT GTC TCT G |
|  | reverse | TGG CTG AGG AAG TC TCT GAG TTG |
| Cathepsin K | forward | TGA GGC TTC TCT TGG TGT CCA TAC |
|  | reverse | AAA GGG TGT CAT TAC TGC GGG |
| NF-ATc1 | forward | AGC TGC ATG GCT ACT TGG AG |
|  | reverse | TTG GTG TTG GAG AGG ATG GC |
| DC-STAMP | forward | TTC GCT CGT CCT GCT TGG |
|  | reverse | GCG GGA TGT CTG GTG ATG TAG |
| OC-STAMP | forward | TGC CCA TCA CGC TCA CG |
|  | reverse | GGC GGA GCT CCC ATT GGT |
| ATP6vOd2 | forward | TCT GAT CGA AAC GCC ATT AGC |
|  | reverse | AAC TTC TGC TGT GAC ATC ACC ATG |
| Beta-actin | forward | GGA CTT CGA GCA AGA GAT GG |
|  | reverse | TGT GTT GGG GTA CAG GTC TTT G |

**Figure S1**. **Expression of IL-20RA and IL-10RB in FLSs from osteoarthritis OA patients.** OA-FLSs were cultured with IL-26 at various concentrations, and the IL-20RA and IL-10RB mRNA levels were determined by real-time PCR. Results are presented as mean ± SEM (n = 3).


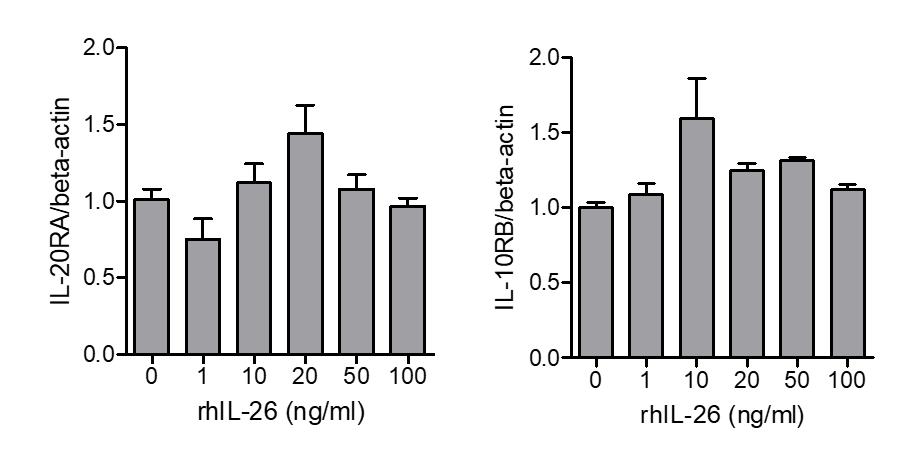


**Figure S2.** **Effect of IL-26 on proinflammatory cytokine levels in FLSs from RA patients.** Following culture of RA synovial fibroblasts with rhIL-26 for 72 h, concentrations of TNF-α, IL-6, and IL-1β in the culture media were determined by sandwich ELISA. Results are presented as mean ± SEM (n = 3).


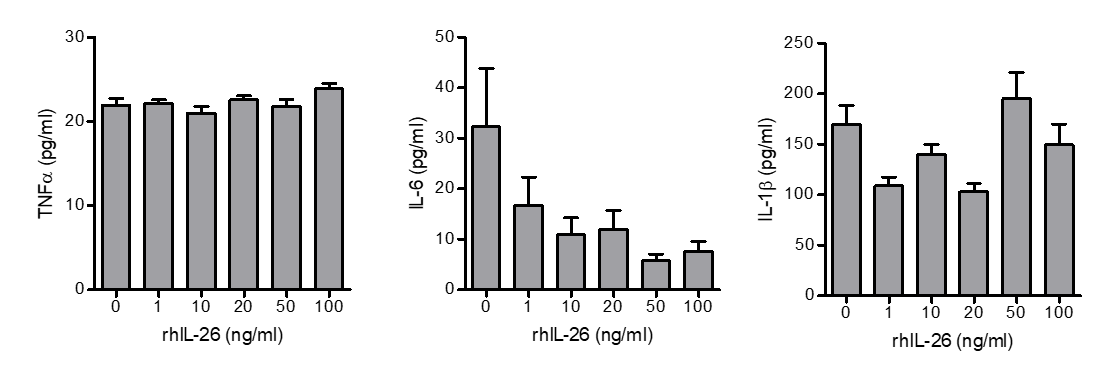


**Figure S3. Expression of IL-20RA and IL-10RB in osteoclasts.** Osteoclasts were cultured with IL-26 at various concentrations, and IL-20RA and IL-10RB mRNA levels were determined by real-time PCR. Results are presented as mean ± SEM (n = 3). ***P < 0.005.


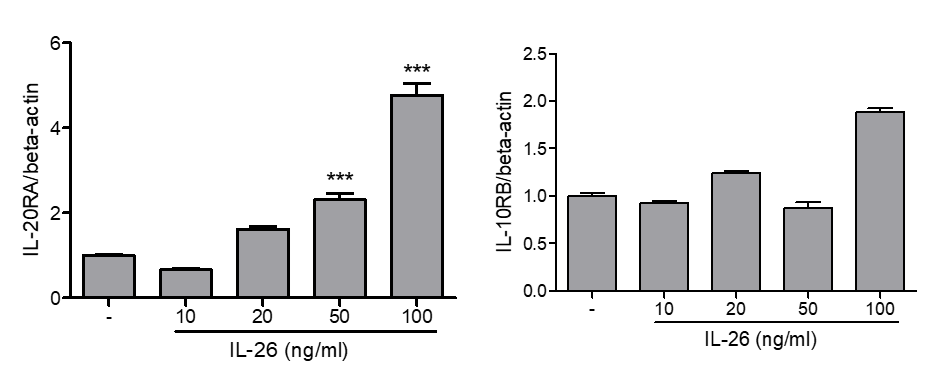


**Figure S4. RANKL concentration in culture media.** RANKL concentration in the culture media without and with 25 ng/mL macrophage colony stimulating factor (M-CSF) was determined by sandwich ELISA. RANKL concentrations in the culture media were also evaluated following incubation with 0–100 ng/mL rhIL-26 for 72 h. The results are presented as mean ± SEM (n = 3).

**
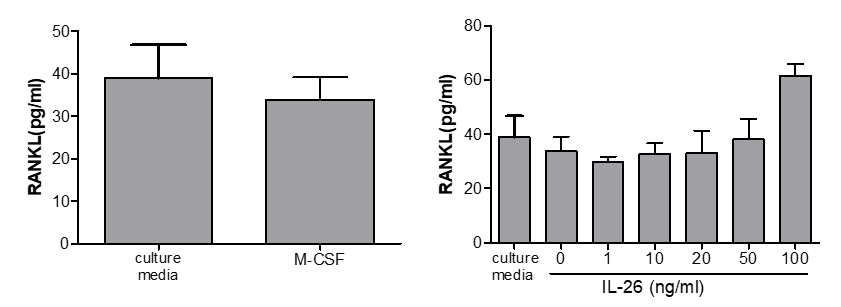
**
